# The Effect of *Lentinus edodes* Stem Powder on the Growth Performance Immune Responses and Gut Microbiota of Sika Deer Fawns

**DOI:** 10.3390/biology15151284

**Published:** 2026-08-04

**Authors:** Chenmin Yu, Yuhang Zhang, Caiyue Zhao, Yichun Yang, Tao Hou, Shukun Zhang, Min Wu, Chongshan Yuan, Aiwu Zhang

**Affiliations:** Key Laboratory of Animal Production, Product Quality and Security, Ministry of Education, Jilin Agricultural University, Changchun 130118, China; 18159056990@163.com (C.Y.); 18343469353@163.com (Y.Z.); 15948838077@163.com (C.Z.); m15141412546_2@163.com (Y.Y.); 18634611920@163.com (T.H.); 18844146800@163.com (S.Z.); koo331500@163.com (M.W.)

**Keywords:** *Lentinus edodes* stem powder, sika deer fawn, growth performance, gut microbiota

## Abstract

As the main byproduct of edible mushroom processing, mushroom stems are often discarded due to their rough texture and poor palatability, resulting in resource waste. However, as a ruminant animal, sika deer has good fiber digestion ability, providing the possibility for the resource utilization of mushroom stems. This study evaluated the effects of dietary supplementation with *Lentinus edodes* stem powder (LESP) on growth performance, nutrient digestibility, serum biochemical parameters, immune responses, and gut microbiota composition in sika deer fawns. A total of 120 weaned fawns (120 ± 10 days old; 35.0 ± 1.0 kg body weight) were randomly assigned to four dietary treatments, receiving a basal diet supplemented with 0% (control), 2.5% (LE1), 5% (LE2), or 7.5% (LE3) LESP for 35 days. Growth performance did not differ significantly among treatments (*p* > 0.05). However, apparent digestibility of ether extract and crude protein was significantly higher in the LE1 group (*p* < 0.05), while LE3 exhibited reduced digestibility of organic matter, ether extract, and crude protein (*p* < 0.05). Serum total protein (TP) and albumin (ALB) levels increased with LESP inclusion, with the highest values observed in LE3 (*p* < 0.05). Serum glucose (GLU) decreased dose-dependently (*p* < 0.05). Immunoglobulin A (IgA), G (IgG), and M (IgM) concentrations were significantly elevated in LE2 and LE3 groups (*p* < 0.05). Gut microbiota analysis revealed that Firmicutes, Bacteroidetes, and Actinobacteria were the dominant phyla. LE3 supplementation altered beta diversity and reduced microbial evenness. At the genus level, LE2 and LE3 increased the abundance of beneficial taxa such as *Planomicrobium* and *Psychrobacter*, while decreasing *Ruminococcus* and *Clostridiaceae_Clostridium*. In conclusion, adding 5% LESP to the diet can increase the digestibility of nutrients, enhance humoral immunity, and regulate the composition of the gut microbiota. This study promotes the transformation of mushroom stems from agricultural waste into functional feed additives, which helps reduce breeding costs, improve animal health, and provide a practical path for the coordinated and sustainable development of the edible mushroom industry and animal husbandry.

## Simple Summary

Large amounts of mushroom stems, a major byproduct of edible mushroom processing, are routinely discarded due to their coarse texture and low edible value, causing massive agricultural resource waste and environmental pressure. Recycling these abandoned mushroom stems as ruminant feed is an eco-friendly and sustainable strategy to realize agricultural waste reutilization, reduce waste accumulation and environmental pollution, and promote the green and coordinated development of the edible mushroom breeding and animal husbandry industries. Sika deer, as typical ruminants with strong fiber digestion capacity, can efficiently utilize mushroom stem resources. This study explored the application value of *Lentinus edodes* stem powder (LESP) in the feeding of weaned sika deer fawns. The results showed that appropriate dietary supplementation with LESP exerted multiple beneficial effects on sika deer fawns without impairing growth performance. Collectively, the addition of LESP in sika deer diet achieves efficient resource recycling of waste mushroom stems, delivers dual benefits of ecological protection and animal health improvement, lowers breeding costs, and provides a feasible sustainable development path for the integrated utilization of agricultural byproducts and healthy deer breeding.

## 1. Introduction

*Lentinula edodes* is a fungus renowned for its medicinal and nutritional properties, characterized by a distinct aroma, palatable taste, and a rich composition of protein, amino acids, and vitamins [1]. This has conferred upon it significant application value in both the food and pharmaceutical sectors. In recent years, rising living standards have increasingly focused consumer attention on the nutritional quality and functional attributes of food. This shift in consumption patterns has catalyzed rapid growth in the edible fungus industry. Concomitantly, this expansion has led to a substantial increase in the generation of processing by-products. The efficient utilization of these by-products has therefore emerged as a critical challenge for the industry’s sustainable development. The stem of *Lentinula edodes* constitutes the primary by-product of mushroom processing. Due to its fibrous texture and inferior palatability compared to the cap, it is frequently discarded as waste. However, accumulating evidence indicates that *Lentinula edodes* stem powder (LESP) is a valuable source of dietary fiber and antioxidant compounds. These properties suggest its potential to enhance the nutritional quality of food products and improve the organoleptic characteristics of animal feed, positioning it as a candidate feed additive [2,3]. Previous study has demonstrated that incorporating appropriate amounts of edible fungus by-products into the diets of ruminants, such as cattle and sheep, can significantly improve feed conversion efficiency, reduce the feed-to-meat ratio, and consequently lower production costs [4,5]. The proposed mechanisms include the stimulation of rumen microbial activity by the high fiber content, leading to enhanced digestive and absorptive capacity, while the associated antioxidant components may bolster animal immunity and reduce disease incidence [6]. Sika deer are economically valuable ruminants, widely distributed in Northeast China. Their antlers, blood, and meat hold considerable medicinal value and are important components in traditional Chinese medicine [7]. As typical herbivorous mammals, sika deer possess a well-developed capacity to digest crude fiber, rendering them well-suited to utilize fiber-rich feedstuffs [8]. Our preliminary investigations revealed that supplementing the diet of sika deer with small amounts of spent *Pleurotus ostreatus* substrate effectively reduces the reliance on concentrated feeds, thereby lowering breeding costs and improving economic returns [9]. Despite these promising findings, research specifically evaluating the application of *Lentinula edodes* stem powder as a feed additive for sika deer fawns remains scarce. Therefore, this study aimed to investigate the effects of graded levels of dietary LESP supplementation on growth performance, serum biochemical indices, and gut microbiota structure in sika deer fawns. By analyzing the response patterns of these indicators, we sought to determine the optimal inclusion level of LESP and evaluate its potential application in herbivore nutrition. This approach not only explores opportunities to diversify the feed resources available for sika deer but also proposes a strategy for valorizing agricultural waste to reduce production costs and improve economic efficiency. The findings are expected to provide a theoretical foundation and technical support for the future integration of edible fungus by-products into livestock feeding systems.

## 2. Materials and Methods

### 2.1. Animals, Diets, and Experimental Design

All experimental procedures were approved by the Institutional Animal Care and Use Committee of Jilin Agricultural University. The experimental animals were sourced from Jilin Changchun Shuangyang Deer Industry Breeding Co., Ltd. (Changchun, China), and experiments were conducted here. A total of 120 weaned sika deer fawns (*Cervus nippon*) aged 120 ± 10 days with an initial body weight of 35.0 ± 1.0 kg were stratified by body weight and sex and randomly assigned to one of four dietary treatments. Each treatment consisted of 30 replicates (pens), with one fawn per pen, and an equal number of males and females per group. Fawns in the control group were fed a basal diet formulated to meet their nutritional requirements. The composition of basal diets is shown in Table 1. According to the previous study [10], fawns in the treatment groups (LE1, LE2, and LE3) received the basal diet supplemented with 2.5%, 5.0%, and 7.5% LESP on a dry matter basis, respectively. The LESP was obtained from a commercial edible mushroom processing facility, air-dried, and ground to pass through a 1 mm screen using a hammer mill prior (Schutte-Buffalo Hammermill, Buffalo, NY, USA) to incorporation into the diets. The nutrient compositions of LESP and basal diets are presented in Table 2. The feeding trial lasted for 35 days, comprising a 5-day adaptation period followed by a 30-day experimental period. Fawns were fed twice daily at 04:00 and 16:00 h. As forage fiber, corn stover was provided ad libitum. The experimental diets (concentrate mixtures containing LESP) were offered only at the 16:00 h feeding to ensure complete consumption. All animals had free access to fresh water throughout the experiment. Deer pens were routinely cleaned and disinfected to maintain hygienic conditions. Individual body weights were recorded at the beginning (day 1) and at the end (day 35) of the experimental period before the morning feeding to determine initial and final body weights. Average daily gain was calculated as the difference between final and initial body weights divided by the number of experimental days.

### 2.2. Sample Collection

During the final five days of the formal feeding trial, fresh fecal samples were collected from excrements naturally discharged by sika deer outside the enclosures. To eliminate interference to gut microbiota composition, the samples were collected immediately after defecation, and only chose the inner intact core portion of feces that had no contact with the ground and external environment. In addition, to reduce individual differences, fresh fecal cores obtained from every 10 individual sika deer within the same experimental group were fully homogenized and blended to form one composite mixed sample for subsequent 16S rRNA sequencing analysis and nutrient digestibility analysis. At the conclusion of the feeding trial, three fawns per treatment were randomly selected for blood collection. Animals were anesthetized by intramuscular injection of 1 mL of xylazine hydrochloride (Taizhou Hoyoo Chemical Co., Ltd., Taizhou, Zhejiang, China) administered via a 1 m blowpipe and hollow dart. Following anesthesia, 10 mL of blood was collected from the jugular vein into vacutainer tubes containing clot activator. Immediately after blood collection, anesthesia was reversed by intramuscular administration of 1 mL of tolazoline hydrochloride (Dayang Chem Co., Ltd., Taizhou, Zhejiang, China) to ensure rapid recovery. Blood samples were allowed to clot at room temperature and then centrifuged at 4000 rpm for 10 min at 4 °C. The resulting serum was aliquoted into 2 mL microcentrifuge tubes (NEST Biotechnology, Wuxi, Jiangsu, China) and stored at −80 °C until subsequent analysis of biochemical parameters and immunoglobulin concentrations.

### 2.3. Digestibility

Crude protein content was determined using the Kjeldahl nitrogen method; ether extract content was quantified by the Soxhlet extraction method; crude ash content was assessed through the ashing method; calcium content was analyzed using the potassium permanganate titration method; total phosphorus content was measured by the molybdenum yellow spectrophotometric method. Fawns were weighed in a fasted state on the first and last day of the experiment in the morning to record initial and final body weights, respectively, which were then used to calculate average daily weight gain. Apparent digestibility is measured using the acid–insoluble ash method. The digestibility of dry matter, organic matter, crude protein, and ether extract is calculated using the following formula:

$$\text{Digestibility}\;\text{of}\;\text{a}\;\text{nutrient}\;(\%)=100*(1-\frac{bc}{ad})$$

a—Content of the nutrient in the feed (%);

b—Content of the nutrient in the feces (%);

c—Content of hydrochloric acid-insoluble ash in the feed (%);

d—Content of insoluble hydrochloric acid in the feces (%).

### 2.4. Serum Biochemical and Immunological Analyses

Serum concentrations of total protein (TP), albumin (ALB), high-density lipoprotein (HDL), low-density lipoprotein (LDL), glucose (GLU), and blood urea nitrogen (BUN) were quantified using an automated biochemistry analyzer (BS-200, Mindray Medical International Ltd., Shenzhen, China). All assays were performed with commercial enzymatic colorimetric kits (Nanjing Jiancheng Bioengineering Institute, Nanjing, China) according to the manufacturer’s protocols. The inter-assay coefficients of variation for all analytes were maintained below 3%. Serum immunoglobulin concentrations, including immunoglobulin A (IgA), immunoglobulin G (IgG), and immunoglobulin M (IgM), were determined using species-specific enzyme-linked immunosorbent assay (ELISA) kits (Nanjing Jiancheng Bioengineering Institute, Nanjing, China). All measurements were performed in duplicate strictly following the manufacturer’s instructions. Optical densities were measured using a microplate reader (Molecular Devices, LLC, San Jose, CA, USA), and immunoglobulin concentrations were calculated from standard curves constructed with reference standards provided in the kits.

### 2.5. Gut Microbiota Analysis

Microbial community composition in fecal samples was characterized by high-throughput sequencing of the bacterial 16S rRNA gene, targeting the hypervariable V3–V4 regions. Total genomic DNA was extracted from 200 mg of fecal sample using the QIAamp DNA Stool Mini Kit (Qiagen, Hilden, Germany) following the manufacturer’s instructions. Mechanical lysis was enhanced by bead-beating using a FastPrep-24 instrument (MP Biomedicals, Santa Ana, CA, USA). The V3–V4 hypervariable regions of the 16S rRNA gene were amplified by polymerase chain reaction (PCR) using universal primers 341F (5′-ACTCCTACGGGAGGCAGCA-3′) and 806R (5′-GGACTACHVGGGTWTCTAAT-3′), which incorporate Illumina adapter sequences. PCR amplification was performed in a 25 μL reaction volume containing 12.5 μL of 2× KAPA HiFi HotStart ReadyMix (Roche, Basel, Switzerland), 1 μM of each primer, and 10 ng of template DNA. Thermal cycling conditions consisted of an initial denaturation at 95 °C for 3 min, followed by 25 cycles of denaturation at 95 °C for 30 s, annealing at 55 °C for 30 s, extension at 72 °C for 30 s, and a final extension at 72 °C for 5 min. PCR products were purified and sequenced on the Illumina MiSeq platform (Illumina, San Diego, CA, USA) using paired-end 300 bp chemistry.

Raw sequencing data were processed using QIIME 2 (version 2019.4) [10]. Demultiplexed sequences were denoised, dereplicated, and filtered for chimeras using the DADA2 plugin (version 2024.5.0) to generate amplicon sequence variants (ASVs). Taxonomic assignment of representative ASV sequences was performed using a pre-trained naive Bayes classifier based on the Greengenes database (version 13_8).

Alpha diversity metrics, including richness (Chao1 index, Observed species), diversity (Shannon and Simpson indices), phylogenetic diversity (Faith’s phylogenetic diversity), evenness (Pielou’s evenness), and sequencing depth (Goods coverage), were calculated based on rarefied ASV tables. Differences in alpha diversity among treatment groups were assessed using Student’s *t*-test. Beta diversity analysis was conducted to evaluate differences in microbial community structure among groups. Principal coordinate analysis (PCoA) and non-metric multidimensional scaling (NMDS) were performed based on weighted and unweighted UniFrac distance matrices. Permutational multivariate analysis of variance (PERMANOVA) with 999 permutations was used to test for significant differences in community composition among groups.

Relative abundances of bacterial taxa were summarized at the phylum and genus levels for each group. Venn diagrams were constructed to visualize unique and shared ASVs among treatment groups. Hierarchical clustering analysis based on Bray–Curtis distances was performed to illustrate similarities in microbial community structure among individual samples. Linear discriminant analysis effect size (LEfSe) was employed to identify differentially abundant bacterial taxa among groups, with a linear discriminant analysis (LDA) score threshold of >5.0.

### 2.6. Statistical Analysis

Growth performance, digestibility, and serum parameters were analyzed by one-way ANOVA followed by Duncan’s multiple range test using SPSS 25.0 (IBM, Armonk, NY, USA). Data are presented as mean ± SD, with statistical significance set at *p* < 0.05. Microbiome bioinformatics was performed using QIIME 2 (2019.4) [11]. Taxonomic classification was conducted using the Greengenes database (http://greengenes.lbl.gov, accessed on 26 June 2025) with a naive Bayes classifier. Alpha diversity indices (Chao1, Observed, Shannon, Simpson, Faith’s PD, Pielou’s evenness, Good’s coverage) were compared between groups using Student’s *t*-test. Beta diversity was assessed using weighted and unweighted UniFrac distances, with differences visualized by PCoA and NMDS. Community composition heatmaps were generated using R (v4.0.3).

## 3. Results

### 3.1. Effect of LESP on Growth Performance of Sika Deer Fawns

According to Table 3, the addition of LESP to the diet did not significantly affect the final weight, body weight gain, or average daily gain of the fawns (*p* > 0.05).

### 3.2. Effect of LESP on Apparent Digestibility in Sika Deer Fawns

As presented in Table 4, dietary LESP supplementation significantly affected nutrient digestibility in sika deer fawns. The apparent digestibility of organic matter was significantly lower in the LE3 group compared to all other groups (*p* < 0.05). Ether extract digestibility was significantly increased in the LE1 and LE2 groups relative to the control and LE3 groups (*p* < 0.05), whereas the LE3 group exhibited a significant reduction compared to the control (*p* < 0.05). Crude protein digestibility was significantly enhanced in the LE1 group (*p* < 0.05) but markedly decreased in the LE3 group relative to the other treatments (*p* < 0.05). No significant differences were observed in dry matter digestibility among groups (*p* > 0.05).

### 3.3. Effect of LESP on Serum Biochemical Indices of Sika Deer Fawns

Dietary LESP supplementation significantly influenced serum biochemical indices in sika deer fawns (Table 5). TP concentrations were significantly elevated in all LESP-supplemented groups compared to the control (*p* < 0.05), with the LE3 group exhibiting the highest levels among treatments (*p* < 0.05). ALB concentration was significantly increased only in the LE3 group relative to the other groups (*p* < 0.05). HDL levels were significantly lower in the LE1 and LE2 groups than in the control (*p* < 0.05), whereas the LE3 group showed a significant increase compared to the control (*p* < 0.05). LDL concentration was significantly higher in the LE3 group than in all other groups (*p* < 0.05). GLU concentrations exhibited a dose-dependent decrease with increasing levels of dietary LESP, with all treatment groups showing significantly lower values than the control (*p* < 0.05). BUN levels were significantly elevated only in the LE1 group compared to the other groups (*p* < 0.05).

### 3.4. Effect of LESP on Immune Indices of Sika Deer Fawns

Dietary LESP supplementation significantly enhanced humoral immunity in sika deer fawns (Table 6). Serum IgG concentrations were significantly increased in all LESP-supplemented groups compared to the control (*p* < 0.05). IgA and IgM levels were significantly elevated in the LE2 and LE3 groups relative to both the control and LE1 groups (*p* < 0.05). These results indicate that LESP supplementation at 5% and 7.5% effectively improves the immune status of fawns.

### 3.5. The Richness and Diversity of Gut Microbiota of Sika Deer Fawns

**Figure 1:**
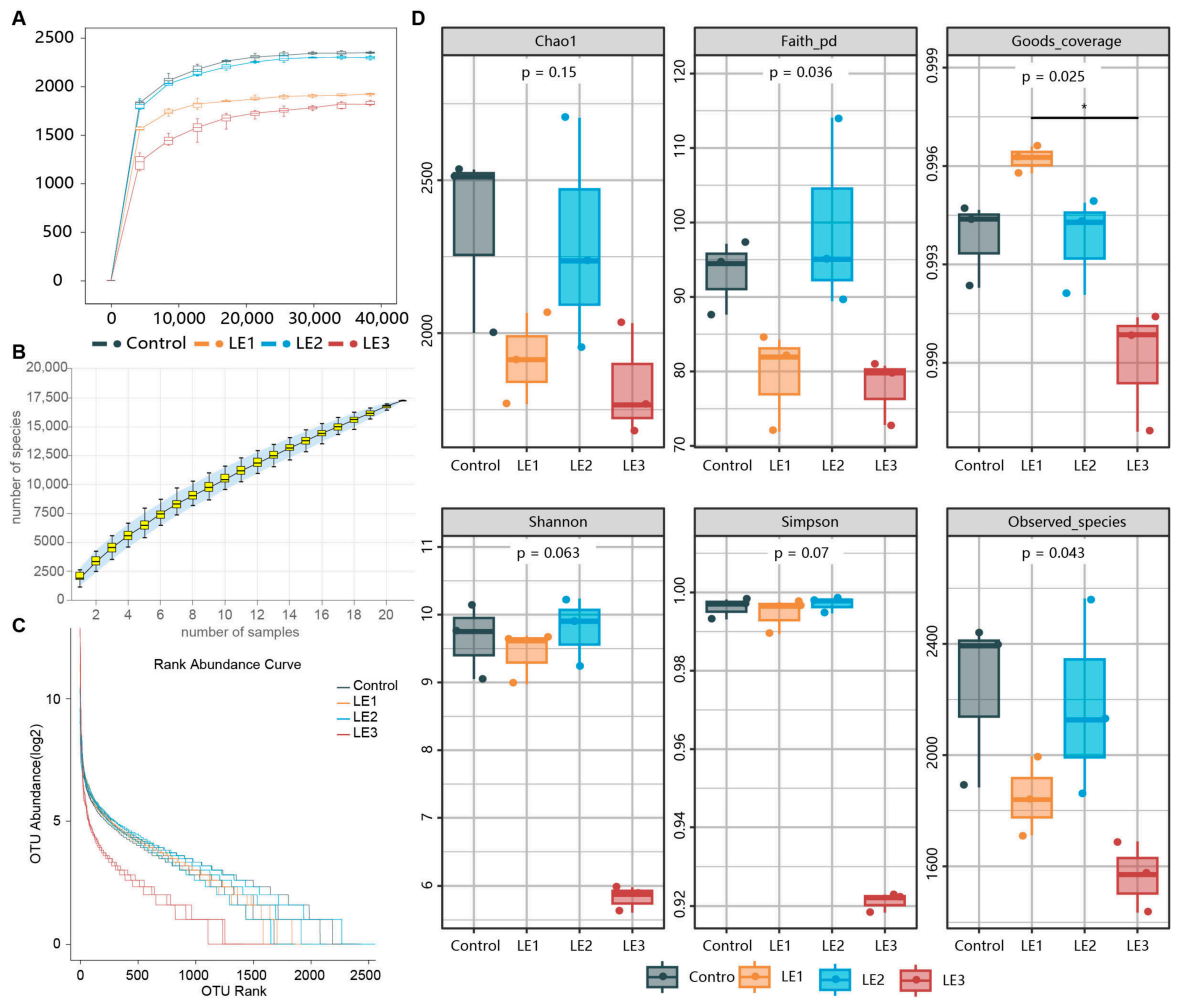
(**A**) Sparse curve of the gut microbiota in sika deer fawns; (**B**) Species accumulation curve of the gut microbiota; (**C**) Rank abundance curve of the gut microbiota in sika deer fawns; (**D**) Alpha diversity indices of the gut microbiota in sika deer fawns. * Indicating significant difference, *p* < 0.05.

As shown in Figure 1A, the horizontal axis represents the flattening depth, and the vertical axis represents the median of the alpha diversity index calculated 10 times. The smoothness of the curve reflects the impact of sequencing depth on the diversity of observed samples. Our results show that the curve is approaching a plateau, indicating that the sequencing results are sufficient to reflect the diversity contained in the current sample. The species accumulation curve can reflect the rate of increase in new species observed during the sample extraction process as the sample size continues to expand. As shown in Figure 1B, with the increase in sample size, the rate of increase becomes flat, indicating that species do not significantly increase with the increase in sample size. Afterwards, the abundance level curve is measured, with the horizontal axis representing the sequence numbers of OTUs arranged by abundance size; The vertical axis represents the abundance value of each OTU in the group. The flatter the line, the smaller the difference in abundance between OTUs in the community, and the higher the uniformity of community composition. Our results showed that the LE3 group had a steep curve, indicating that high concentrations of LESP in the diet reduced the uniformity of gut microbiota (Figure 1C). Alpha diversity is shown in Figure 1D, the addition of LESP in the diets significantly changed Faith’s PD, Good’s coverage, and Observed species (*p* < 0.05). Among them, Good’s coverage index of LE3 was significantly decreased compared to the LE1 group (*p* < 0.05).

### 3.6. Differences in Gut Microbiota of the Gut Microbiota in Sika Deer Fawns

**Figure 2:**
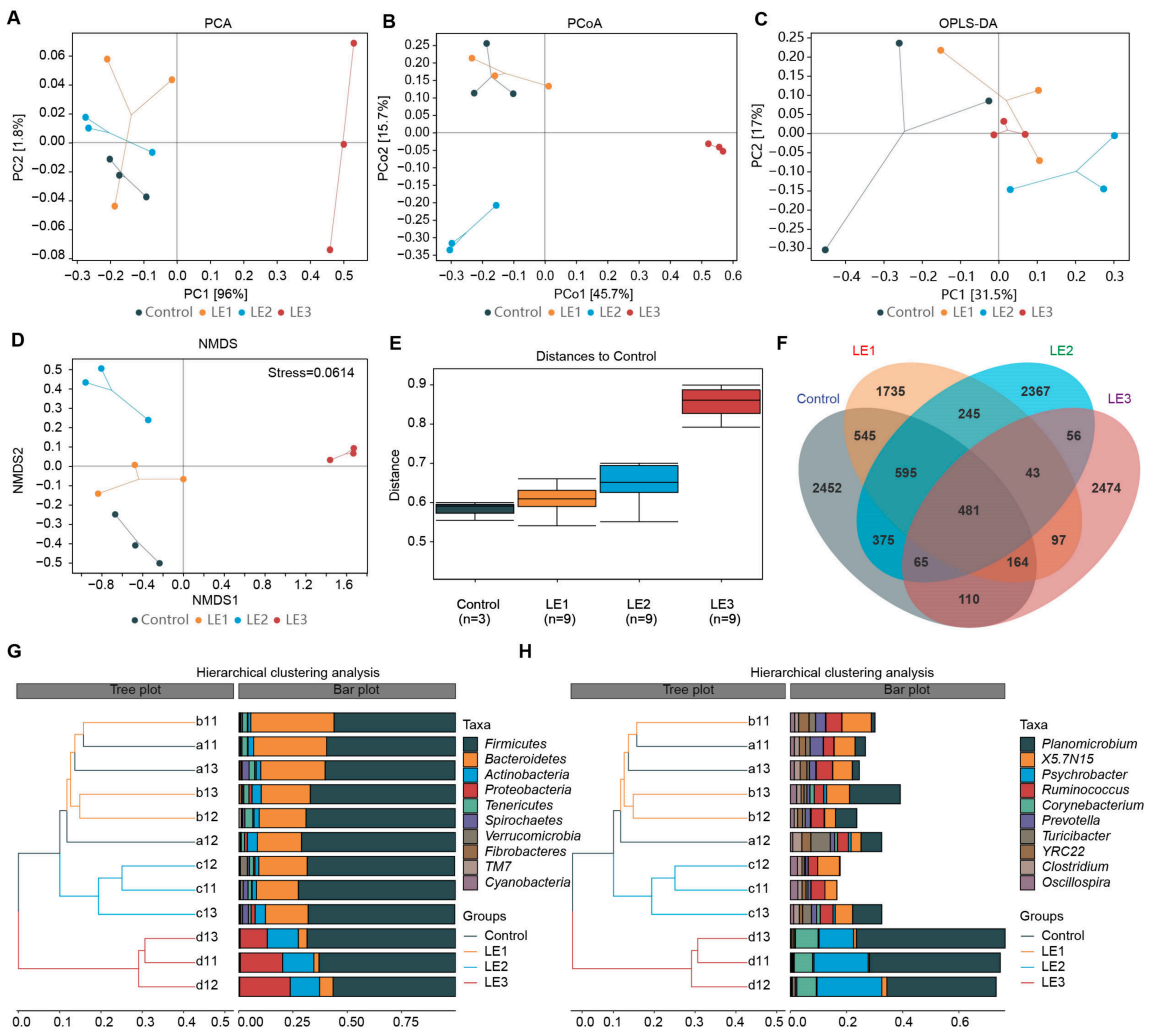
(**A**) PCA; (**B**) PCoA; (**C**) OPLS-DA; (**D**) NMDS (**E**) Distances among the groups; (**F**) OTU-based venn diagram; (**G**) Hierarchical clustering tree diagram of phylum level; (**H**) Hierarchical clustering tree diagram of genus level. Values sharing different lowercase letters indicate statistically significant differences (*p* < 0.05).

Beta diversity examined the comparison of diversity between different habitats, specifically, the differences between samples. In order to detect the beta diversity of gut microbiota, we conducted PCA and NMDS analysis on each group of samples. As shown in Figure 2A–D, the distance between the control group, LE1 group, and LE2 group is relatively close, indicating that the microbial community structure among the three groups is similar. The distance between LE3 and other groups is relatively far, indicating that high-dose LESP in the diet can alter the microbial community structure of feces. Afterwards, we analyzed the differences between the groups. Figure 2E shows the distribution box plots of the distances between samples in this group and the box plots of the distances between samples in this group and the other groups. The results showed significant differences between the LE3 group and the control group, while the differences between the LE1 and LE2 groups were relatively small compared to the control group. The Venn diagram (Figure 2F) analyzes the number of common and unique OTUs among the different groups of sika deer fawns. In total, the four groups share 481 OTUs. The control group possesses 2452 unique OTUs, the LE1 group contains 1735 unique OTUs, the LE2 group holds 2367 unique OTUs, and the LE3 group has 2474 unique OTUs. The similarity of each group of samples is displayed by hierarchical clustering trees. The panel on the left is a hierarchical clustering tree diagram, where samples are clustered based on their similarity to each other. The shorter the branch length between samples, the more similar the two samples are; The panel on the right is a stacked bar chart of the top 10 genera in terms of abundance. Figure 2G and Figure 2H, respectively, show the microbiota of phyla and genera in the gut. The results showed that samples from the control group (a11, a12, a13), LE1 group (b11, b12, b13), and LE2 (c11, c12, c13) were clustered together, while the LE3 group (d11, d12, b13) was located on a single branch. Indicating that with the increase in LESP levels, the differences in gut microbiota also increase.

### 3.7. Community Composition and Abundance of Gut Microbiota

**Figure 3:**
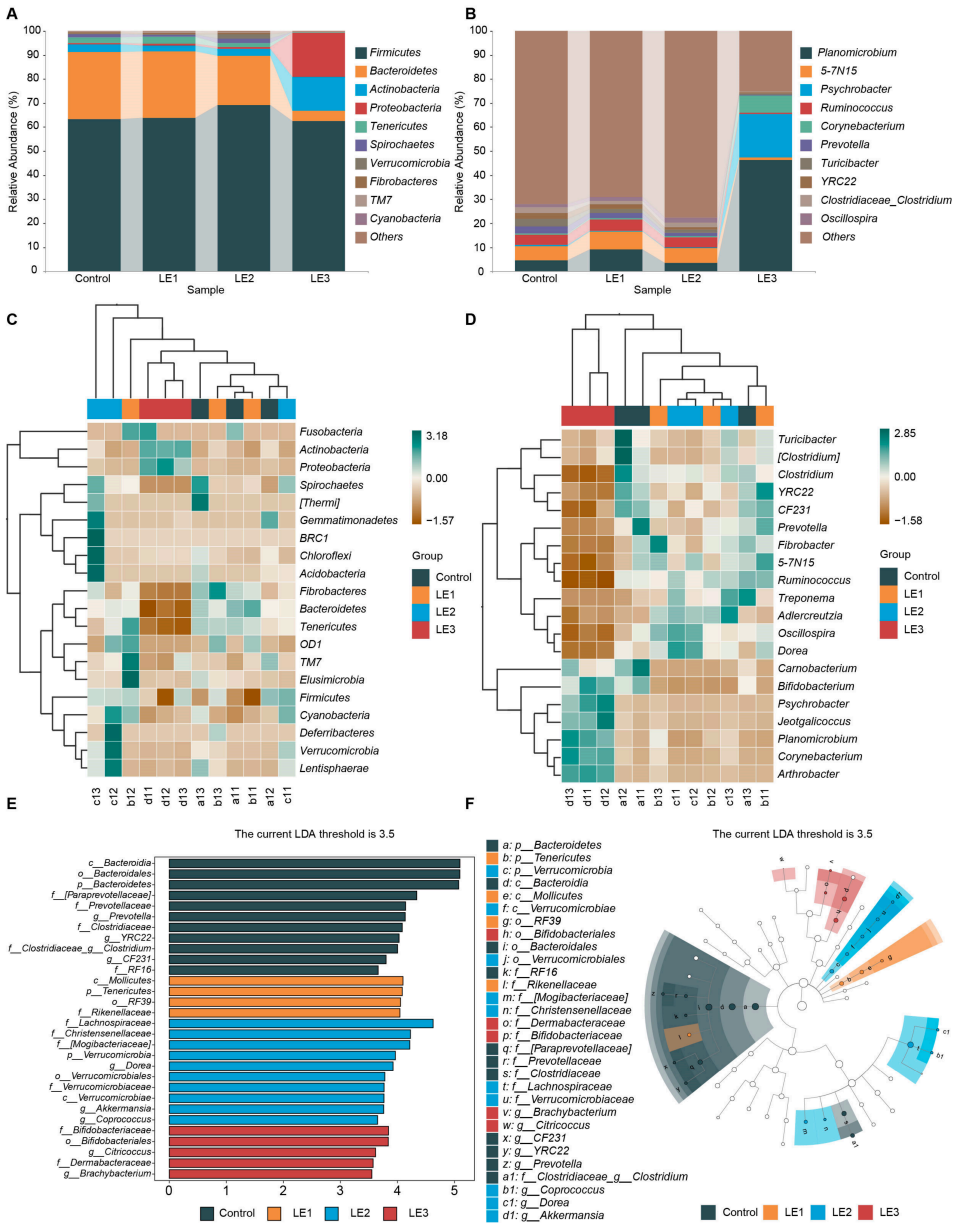
(**A**) Gut microbiota composition of sika deer fawns at the phylum level; (**B**) Gut microbiota composition of sika deer fawns at the genus level; (**C**) Heatmap of phylum level species composition of gut microbiota; (**D**) Heatmap of genus level species composition of gut microbiota; (**E**) LDA Score of different species. (**F**) Cladogram of different species.

The top ten major bacterial phylum and genus in the feces of sika deer fowns are shown in Figure 3A and Figure 3B, respectively. Firmicutes, Bacteroidetes, Actinobacteria, Proteobacteria, Tenericutes, Spirochaetes, Verrucomicrobia, Fibrobacteres, TM7, and Cyanobacteria were the main phylum in the feces (Figure 3A). *Planomicrobium*, 5-*7N15*, *Psychrobacter*, *Ruminococcus*, *Corynebacterium*, *Prevotella*, *Turicibacter*, *YRC22*, *Clostridiaceae_Clostridium*, and *Oscillospi* were the main genus in the feces (Figure 3B). From the results, it was found that there were significant changes in the gut microbiota of the LE3 group compared to other groups. To more comprehensively elucidate the inter-sample variations in species composition and to vividly depict the trends in abundance distribution among samples, we generated a heatmap of species composition in Figure 3C,D. This heatmap was constructed by leveraging the abundance data of the top 20 phyla, which were ranked according to their average abundance. Compared to the control group, the Bacteroidetes in the LE3 group decreased from 27.83% to 4.2%; Actinobacteria increased from 3.1% to 14.06%; Proteobacteria increased from 0.76% to 18.44% in the phylum level (Figure 3C). In the genus level, *Planomicrobium* in the LE3 group increased from 4.52% to 46.18%; *5-7N15* decreased from 5.98% to 1.07%; *Psychrobacter* increased from 0.4% to 18.24%; *Ruminococcus* decreased from 4.42% to 0.37%; *Corynebacterium* increased from 0.44% to 7.17% compared to the control group (Figure 3D). To determine the difference in the degree of bacterial enrichment between the groups, we performed a linear discriminant analysis effect size (LEfSe) based on LDA scores > 5 (Figure 3E,F). The results demonstrated that in the genus level, *Prevotella*, *YRC22*, and *CF231* were enriched in the control group; *Dorea*, *Akkermansia*, and *Coprococcus* were enriched in the LE2 group; *Citricoccus* and *Brachybacterium* were enriched in the LE3 group.

## 4. Discussion

Sika deer represent an economically valuable livestock species whose health status directly influences production efficiency and economic returns. The strategic inclusion of functional feed additives offers a promising approach to enhance growth performance and immunological competence in these animals. Despite its abundance of bioactive polysaccharides and B vitamins, LESP has historically been underutilized and largely discarded as agricultural waste, resulting from its fibrous texture and poor palatability [12,13]. The present study systematically evaluated the effects of graded LESP supplementation on growth performance, nutrient digestibility, serum biochemistry, and gut microbiota composition in sika deer fawns, with the objective of determining its potential as a functional feed ingredient. Our findings demonstrate that dietary inclusion of 5% LESP optimizes protein and fat digestibility, enhances humoral immune responses, and preserves gut microbial homeostasis in sika deer fawns.

LESP contains considerable levels of protein and various bioactive constituents, including polysaccharides [14]. Body weight gain and average daily gain are fundamental indicators reflecting the growth performance and general health status of fawns [15]. In the present study, dietary inclusion of 2.5% and 5% LESP exhibited a numerical tendency to improve body weight gain and average daily gain, with the 5% supplementation level yielding the most favorable growth outcomes. These observations align with previous findings in other animal models. It is reported that partially replacing the basal diet with fermented mushroom-supplemented feed in growing-finishing Berkshire pigs increased final body weights by 30%, 50%, and 70% compared to controls [16]. Similarly, studies investigating shiitake stem residue powder, mushroom stem powder, and fermented enoki mushroom bran have demonstrated significant enhancements in final body weight and average daily gain across various livestock species [17,18,19], consistent with the trends observed in our experiment. It is noteworthy that the present trial was conducted during winter, when low ambient temperatures increase maintenance energy requirements. Under such conditions, a greater proportion of absorbed nutrients is prioritized for basal metabolic functions rather than growth, potentially attenuating the magnitude of treatment effects [20]. This may account for the absence of statistically significant differences in growth parameters despite the observed numerical improvements. In addition, the feeding trial lasted only 35 days, which may not be sufficient to determine whether the observed effects can be maintained over a longer production period. Collectively, these findings suggest that moderate LESP supplementation, particularly at 5%, possesses the potential to enhance growth performance in sika deer fawns. Longer trials would be useful to better assess growth performance, health status, and the real economic value of this supplementation strategy.

Dietary LESP supplementation exerted positive effects on nutrient digestibility in sika deer fawns. A prior study demonstrated partial dietary replacement with mushroom-derived products not only improved growth performance in Yanbian cattle but also enhanced economic returns and promoted better health status [21]. Similarly, supplementation with fermented enoki mushroom bran significantly increased the digestibility of dry matter, crude protein, and ether extract in Guanzhong dairy goats [22]. Furthermore, inclusion of 30% fermented king oyster mushroom bran in the basal diet of Guizhou black goats markedly improved dry matter and crude protein digestibility [23]. In the present study, crude protein and ether extract digestibility were significantly improved in fawns receiving a small amount of LESP supplementation, consistent with these earlier reports. These studies suggest that moderate LESP supplementation optimizes nutrient digestibility, whereas excessive inclusion may exert deleterious effects, potentially due to the high fiber content of LESP overwhelming the digestive capacity of young ruminants with incompletely developed rumen function. Follow-up research will conduct full quantitative detection of functional active components in LESP. Moreover, animal preference trials will be arranged to evaluate feed palatability and a complete input–output economic model will be established to quantify the economic return of LESP supplementation.

Dietary LESP supplementation significantly modulated serum biochemical indices in sika deer fawns in a dose-dependent manner [5,24]. TP and ALB concentrations increased across all treatment groups, with the highest levels observed in LE3, indicating enhanced protein metabolism and nitrogen retention [25]. Lipid metabolism exhibited differential responses to LESP inclusion. Supplementation with 2.5% and 5% LESP reduced HDL concentrations, whereas 7.5% LESP elevated both HDL and LDL levels. The lower LDL in LE1 and LE2 groups suggests potential cardiovascular benefits, while elevated LDL in LE3 indicates adverse effects on cholesterol homeostasis at excessive inclusion [26]. GLU decreased progressively with increasing LESP levels, reflecting enhanced glucose utilization, consistent with previous reports on mushroom polysaccharides [25]. BUN was elevated only in LE1, while LE2 and LE3 showed numerically lower values compared to controls, suggesting improved nitrogen utilization efficiency at higher inclusion levels [27]. Collectively, moderate LESP supplementation, particularly at 5%, optimizes protein and energy metabolism in sika deer fawns.

The immunomodulatory properties of LESP represent one of its most significant functional attributes in animal nutrition. Immunoglobulins, including IgA, IgM, and IgG, serve as critical effectors of humoral immunity, providing defense against pathogenic microorganisms [28,29,30]. Lentinula edodes polysaccharides, particularly β-(1,3)-D-glucans, are well-recognized for their immune-enhancing activities, including stimulation of macrophage activation, lymphocyte proliferation, and antibody production [31,32,33]. In the present study, dietary LESP supplementation significantly elevated serum immunoglobulin concentrations in sika deer fawns. Immunoglobulin G levels were increased across all treatment groups compared to controls, while IgA and IgM concentrations were significantly enhanced only in fawns receiving 5% and 7.5% LESP. These findings align with previous reports demonstrating that mushroom-based supplements augment humoral immunity in various animal species [34]. Notably, the enhanced immunoglobulin production at moderate LESP inclusion suggests improved immune competence, which may confer greater resistance to infectious challenges and contribute to overall health maintenance in sika deer fawns. Although 5% and 7.5% LESP improved immune competence, this higher dose negatively affected the digestibility of organic matter, ether extract, and crude protein. This suggests that the beneficial range of LESP supplementation may be relatively narrow.

The beneficial effects of LESP on growth performance, nutrient digestibility, and immune function may be mediated, at least in part, through modulation of the gut microbiota. The gut microbial community plays a pivotal role in nutrient digestion, immune system development, and maintenance of host physiological homeostasis [35]. Bacteria establish a symbiotic relationship with the host, and their compositional structure and metabolic byproducts actively regulate metabolic processes and immune function [36]. In the present study, 16S rRNA gene sequencing revealed that dietary LESP supplementation, particularly at the 5% inclusion level, significantly altered the richness and diversity of the fecal microbiota in sika deer fawns. Analysis of alpha diversity indices demonstrated that LESP supplementation modulated microbial community structure, with the 5% treatment group exhibiting optimal balance between species richness and evenness. Beta diversity analysis further revealed distinct clustering of microbial communities among treatment groups, indicating that LESP supplementation induced shifts in overall microbial compositional structure.

The major bacterial phyla in the intestines of young deer include Firmicutes, Bacteroidetes, Actinobacteria, and Proteobacteria. Among them, Firmicutes and Bacteroidetes account for approximately 85% and play pivotal roles in energy absorption and gut immunity. The addition of 5% LESP significantly increased the abundance of beneficial bacteria involved in cellulose decomposition and nitrogen metabolism. On the contrary, when the LESP addition was increased to 7.5%, the abundance of beneficial bacteria decreased, while the abundance of potentially harmful bacteria such as *Actinomyces*, *psychrophilic* bacteria, and spore-forming bacteria increased significantly. This could have an adverse impact on immunity and digestive function [37]. Therefore, a supplementation level of 5% is more conducive to optimizing the gut bacterial community structure and enhancing nutritional utilization efficiency and supporting normal growth.

At the genus level, LESP supplementation modulated the abundance of specific bacterial taxa with known functional roles. The 5% treatment group exhibited increased relative abundances of cellulolytic bacteria (*Ruminococcus*, *Fibrobacter*) and saccharolytic fermenters (*Prevotella*), which are implicated in fiber degradation and volatile fatty acid production [38,39]. Concurrently, potentially pathogenic genera including *Clostridium* and *Enterococcus* were reduced in the 5% LESP group compared to controls [40,41]. These compositional shifts suggest that moderate LESP supplementation promotes a microbial community structure conducive to enhanced nutrient utilization and improved gut health. Notably, the 7.5% LESP treatment induced more pronounced alterations in microbial composition, including significant increases in potentially deleterious taxa such as *Psychrobacter* and *Corynebacterium* [42,43], alongside reductions in beneficial genera including *Prevotella* and *Ruminococcus* [44,45]. This dysbiotic pattern may underlie the impaired nutrient digestibility and attenuated immune responses observed at the highest supplementation level, highlighting the importance of dose optimization when incorporating LESP into fawn diets. In this study, we only performed 16S rRNA gene sequencing to characterize the alteration in gut bacterial community structure, without confirming their functional consequences. Additional analyses, such as short-chain fatty acid measurement, metagenomics, or metabolomics, would help determine whether these microbial changes are directly linked to improved gut function or immunity.

The limitations of the present study need to be further emphasized. Although LESP supplementation can improve nutrient digestibility, serum biochemical indices, immunoglobulin levels and gut microbiota composition, its effects on productive performance remain to be confirmed. LESP contains a variety of potential active ingredients such as dietary fiber and polysaccharides, yet the mechanisms underlying the observed effects were not fully clarified. This experiment was conducted only in weaned sika deer fawns under specific feeding and management conditions, and its effects on adult sika deer and other ruminants need to be further investigated.

## 5. Conclusions

This study demonstrates that dietary supplementation with 5% LESP optimizes nutrient digestibility, enhances immune function, and maintains gut microbiota homeostasis in sika deer fawns without adversely affecting growth performance. Higher inclusion (7.5%) impairs nutrient absorption and reduces microbial diversity. These findings support LESP as a sustainable functional feed additive for deer nutrition, promoting agricultural by-product valorization. Further research is needed to evaluate long-term effects and optimize application strategies.

## Figures and Tables

**Table 1:** Composition of Basal Diets (Dry matter basis, %).

Item	Content
Corn	59.00
Soybean Meal	30.00
Corn Germ Meal	5.00
Cane Molasses	1.00
Sodium Chloride	2.00
Limestone	1.00
Minerals	1.00
Vitamins	1.00
Total	100.00

**Table 2:** Nutrient Content of Basal Diets and LESP (Dry matter basis, %).

Item	LESP	Control	LE1	LE2	LE3
Moisture	10.53	13.51	13.44	13.36	13.29
Crude Protein	13.72	18.80	18.67	18.55	18.42
Ether Extract	11.63	2.52	2.75	2.98	3.20
Crude Ash	4.65	7.37	7.30	7.23	7.17
Calcium	0.63	1.83	1.80	1.77	1.74
Total Phosphorus	1.93	0.55	0.58	0.62	0.65

**Table 3:** Effects of LESP on Growth Performance of Sika Deer Fawns.

Item	Control	LE1	LE2	LE3
Initial Weight (kg)	34.90 ± 1.56	34.17 ± 1.27	36.02 ± 0.18	36.18 ± 0.08
Final Weight (kg)	43.00 ± 0.92	42.27 ± 1.10	42.70 ± 1.71	41.43 ± 1.19
Body Weight Gain (kg)	8.10 ± 0.72	8.10 ± 0.56	8.43 ± 0.57	7.40 ± 0.66
Average Daily Gain (kg)	0.23 ± 0.03	0.24 ± 0.01	0.24 ± 0.02	0.21 ± 0.03
Dry matter intake (kg·d^−1^)	1.46 ± 0.16	1.48 ± 0.05	1.47 ± 0.07	1.39 ± 0.16
feed-to-gain ratio	6.33 ± 0.64	6.09 ± 0.07	6.09 ± 0.31	6.61 ± 0.62

Control: Basic diet + 0% Lentinula edodes stem powder; LE1: Basic diet + 2.5% Lentinula edodes stem powder; LE2: Basic diet + 5%; LE3: Basic diet + 7.5%.

**Table 4:** Effects of LESP on apparent digestibility (%) in sika deer fawns.

Item	Control	LE1	LE2	LE3
Dry matter digestibility	70.06 ± 1.73	71.22 ± 1.85	71.97 ± 1.91	70.11 ± 0.82
Organic matter digestibility	70.84 ± 1.43 ^a^	71.36 ± 1.94 ^a^	70.96 ± 1.11 ^a^	67.09 ± 2.14 ^b^
Ether extract digestibility	62.89 ± 1.56 ^b^	67.07 ± 0.81 ^a^	67.16 ± 0.33 ^a^	60.73 ± 0.58 ^c^
Crude protein digestibility	69.88 ± 0.48 ^b^	73.48 ± 0.74 ^a^	70.32 ± 1.16 ^b^	67.16 ± 1.04 ^c^

Control: Basic diet + 0% Lentinula edodes stem powder; LE1: Basic diet + 2.5% Lentinula edodes stem powder; LE2: Basic diet + 5%; LE3: Basic diet + 7.5%. Data in the same row without letters or with the same lowercase letters indicate no significant difference (*p* > 0.05); different lowercase letters indicate significant differences (*p* < 0.05).

**Table 5:** Effect of LESP on Serum Biochemical Indices of Sika Deer Fawns.

	Control	LE1	LE2	LE3
Total Protein (TP, g/L)	44.03 ± 0.31 ^c^	50.57 ± 4.05 ^b^	51.07 ± 1.53 ^b^	57.58 ± 0.40 ^a^
Albumin (ALB, g/L)	23.60 ± 0.17 ^b^	26.83 ± 2.07 ^b^	27.30 ± 0.53 ^b^	31.87 ± 0.12 ^a^
High-density lipoprotein (HDL, mmol/L)	1.21 ± 0.05 ^b^	1.10 ± 0.02 ^c^	0.94 ± 0.06 ^d^	1.32 ± 0.01 ^a^
Low-density lipoprotein (LDL, mmol/L)	0.18 ± 0.03 ^b^	0.20 ± 0.03 ^b^	0.17 ± 0.01 ^b^	0.26 ± 0.01 ^a^
Blood glucose (GLU, mmol/L)	7.23 ± 0.38 ^a^	5.29 ± 0.19 ^b^	4.85 ± 0.23 ^c^	3.76 ± 0.03 ^d^
Blood urea nitrogen (BUN, mmol/L)	9.46 ± 0.39 ^b^	11.31 ± 1.18 ^a^	9.35 ± 0.03 ^b^	9.24 ± 0.05 ^b^

Control: Basic diet + 0% Lentinula edodes stem powder; LE1: Basic diet + 2.5% Lentinula edodes stem powder; LE2: Basic diet + 5%; LE3: Basic diet + 7.5%. Data in the same row without letters or with the same lowercase letters indicate no significant difference (*p* > 0.05); different lowercase letters indicate significant differences (*p* < 0.05).

**Table 6:** Effect of LESP on Immune Indices of Sika Deer Fawns.

Items	Control	LE1	LE2	LE3
Immunoglobulin A (IgA, mg/L)	52.04 ± 6.74 ^b^	61.39 ± 8.01 ^b^	73.92 ± 6.46 ^a^	71.53 ± 10.42 ^a^
Immunoglobulin G (IgG, g/L)	1.99 ± 0.76 ^b^	2.49 ± 0.21 ^a^	2.53 ± 0.40 ^a^	2.36 ± 0.13 ^a^
Immunoglobulin M (IgM, mg/L)	2.53 ± 0.31 ^b^	2.81 ± 0.37 ^b^	3.21 ± 0.49 ^a^	3.08 ± 0.56 ^a^

Control: Basic diet + 0% Lentinula edodes stem powder; LE1: Basic diet + 2.5% Lentinula edodes stem powder; LE2: Basic diet + 5%; LE3: Basic diet + 7.5%. Data in the same row without letters or with the same lowercase letters indicate no significant difference (*p* > 0.05); different lowercase letters indicate significant differences (*p* < 0.05).

## Data Availability

The dataset generated and/or analyzed during the current study is available from the corresponding authors on reasonable request.
